# Clinical Proteomics of Breast Cancer

**DOI:** 10.2174/138920210793175930

**Published:** 2010-11

**Authors:** Y. Baskın, T. Yiğitbaşı

**Affiliations:** 1 Dokuz Eylul University, Institute of Oncology, 35340 Inciraltı, Izmir, Turkey; 2 Clinical Biochemistry Clinic, Ataturk Training and Research Hospital, 35360, Izmir, Turkey

**Keywords:** Breast cancer, early diagnosis, prognostic markers, proteomic techniques, micro array techniques, mass spectrometry, surface enhanced laser desorption ionisation, matrix-assisted laser desorption ionisation.

## Abstract

Despite the lifetimes that increased in breast cancers due to the the early screening programs and new therapeutic strategies, many cases still are being lost due to the metastatic relapses. For this reason, new approaches such as the proteomic techniques have currently become the prime objectives of breast cancer researches. Various omic-based techniques have been applied with increasing success to the molecular characterisation of breast tumours, which have resulted in a more detailed classification scheme and have produced clinical diagnostic tests that have been applied to both the prognosis and the prediction of outcome to the treatment. Implementation of the proteomics-based techniques is also seen as crucial if we are to develop a systems biology approach in the discovery of biomarkers of the early diagnosis, prognosis and prediction of the outcome of the breast cancer therapies. In this review, we discuss the studies that have been conducted thus far, for the discovery of diagnostic, prognostic and predictive biomarkers, and evaluate the potential of the discriminating proteins identified in this research for clinical use as breast cancer biomarkers.

## 1 INTRODUCTION

Breast cancer is the most commonly encountered cancer in our country, as it is in the world [1, 2]. Breast cancer occurs in four stages. While the cases in the places where the preventive medicine is widespread and/or can benefit from the developed medical technologies, and receive diagnosis in the first or second stage, the others receive diagnosis in the third and fourth stages. Only half of the cancer cases that receive diagnosis are within the localized period [3]. 

Although not withstanding promising progress has been made by the application of the screening programs and the systemic therapies in the breast cancer cases, there are still many patients dying following the metastatic relapse. This fact brings new approaches in breast cancer researches to the agenda. What this means in clinical terms is that under today’s therapeutic conditions, these early diagnosed cases have approximately a nine times greater expectation of remaining healthy for 10 years compared to those of advanced periods [4].

Breast cancer is a complex disease which can gain a more invasive and resistant character by numerous molecular changes that bring about cell proliferation and genetic instability. This heterogenity creates different subgroups in molecular level and causes different clinical results and therapeutical responses. Studies that are done for the purpose of reaching better clinical results, have been focused on determining the molecular structures which are the causes of the disease, the cancer markers and the molecular structures that can constitute new therapeutical objectives, which can follow up the early diagnosis of the disease, therapeutical response and the relapses after the therapy by setting forth the stages of the disease and the differences that are peculiar to disease and person [5]. This approach, which requires the observation of the cellular changes simultaneously, has reflected in the clinical studies only by the developing technology, within the completion process of the genom analysis. The genom studies have enabled us to understand the molecular fundamentals of the diseases. However, rapidly changing cellular functions have clarified through proteome studies because the same genom may give rise to different proteome outputs [6]. While the term “proteome” was first used in 1994 and refers to the collection of proteins of one cell, tissue or organism, the “proteomic” refers to the studies related with all the biological activities of the proteome. In cancer researches, the protemoic technologies produce very valuable data, differentiating and describing functional and organisational medical pathways [7], determining the molecular structure which is the cause of the disease in tissue and biological liquids, the stages of the disease or the differences peculiar to the disease and the patient [8]. In recent years, the proteome studies that are done in various biological samples have increased. Numerous preliminary studies are being performed on tumour tissue and biological fluids (serum, needle aspiration fluid, ductal lavage fluid and tumour intercellular fluid) for the purpose of understanding the disease processes better in the breast cancer, too. 

In this review, the clinical applications of the proteomic methods in breast cancer cases will be evaluated.

## 2 PROTEOMIC METHODS

In clinical studies, certain methods have come into prominence in terms of its applicability in the field. The methods of determination of known proteins either multiply or as multiple samples simultaneously, form the basis of micro array techniques. In this field, micro arrays of tissues are used which measure a known protein sequence simultaneously in a large number of tumour tissue samples. The studies which started at the level of DNA, mRNA, were first used for immunohistochemistry at the protein level in 1998. None the less, there are about 1500 publications in the field of cancer. [TMA is so far the most used proteomic technique in oncology whith more than 4,600 publications in PubMed in November 2009 which involves in the terms of “tissue microarrays” and “cancer”].

The most important advantage of this method is its ability to evaluate a large number of samples under the same conditions simultaneously, and have the ability to perform analysis with both paraffin block and frozen tissue samples. It is also useful from the point of tissue archiving. In this area another technique which has just started to be used is the forward and reverse phase protein microarray method, which has made it possible to work with tissue lysates, interstitial fluids and serum samples. However, the limiting aspect in all these array methods is that, the analyses are limited to those performed with known antibodies [9]. 

The methods which can define and measure large numbers of unknown proteins are the techniques based on mass spectrometric (MS). After a chemical or physical separation, a large number of protein markers defining the tumour phenotype may be measured. Mass spectrometry based techniques require substantial sample preparation prior to analysis. The immune affinity, 2- dimensional gel electrophoresis (2DE), and free flow electrophoresis (FFE) are the most widely used methods [10]. In the traditional method of 2DE proteins are separated from a complex mixture according to electrical charge and dimensional differences. The advantage of this method is that large numbers (3.000-10.000) of proteins can be separated visually. The technique was first described by O’Farrel in 1975 [11]. The first 2-dimensional gel based proteomic database was created in 1981 [12]. The problems of repeatability and standardisation associated with the method were overcome with the use of the Immobilised pH gradient (IPG) [13]. The development of the differential in gel electrophoresis (DIGE) technique, in which proteins from different sources are marked with fluorescent dyes, separated with 2DE and defined by MS, increased the use of the method especially in cancer research. However, difficulties continue with the visualisation of proteins in very small amounts or of very large or very small dimensions. It is still widely used because it makes functional proteomic and antibody studies possible, and because of its superiority in the definition of unknown proteins [14]. In spite of this, methods of analysis which do not rely on a gel substrate are used more frequently in clinical studies. Liquid chromatography (two-dimenisonal liquid-phase separation together with IEF) and its joint use with isotope-coded affinity tags (ICAT) providing sample tagging at source, the possibility of multidimensional protein analysis afforded by multidimensional protein identification technology (MudPIT) HPLC-cation exchange and reverse phase partnered with (MS/MS), provides the required quick and easy sample preparation for clinical samples [15-18]. 

In Mass spectroscopy which has begun to be used in recent years, the analysis of complex protein samples is performed by the determination of the mass/charge ratio (m/z) and the number of ions for each m/z value of a pressurised gas phase ion mixture. A mass spectrometer consists of an ionisation source, a mass analyser and a detector. In protein biochemistry two ionisation techniques are used predominantly: Matrix-assisted laser desorption ionisation (MALDI) for the analysis of simple peptide structures, and electrospray ionisation (ESI) for more complex samples. However, because it enables analysis of more complex samples, surface enhanced laser desorption ionisation (SELDI) a development of the MALDI technique, is widely used especially in cancer proteomics. In this method protein wafers with a choice of different surfaces are used for protein purification, analysis and molecular reactions.

Four types of mass analysers are used commonly in proteomic studies: ion trap, time of flight (TOF), quadrupole, and Fourier transform ion cyclotron (FT-MS). For analysis of the mass spectrum, statistical and bioinformatics tools are needed,some commercially available examples of them are Proteome, Quest, Propek, Bamf, and Biomarker Wizard [19, 20].

In literature, attention is drawn to two important topics regarding SELDI-TOF-MS analyses. The first of these is providing a selection process prior to data analysis of the spectra which has poor quality and the lack of analytical validity as well as the quality control procedures that are performed for this purpose. This procedure will reduce noise in the proteomic data, and will be reflected as lower variability in diagnostic validity [21]. Another problem discussed is in terms of diagnostic validity. The protein pattern data with SELDI-TOF MS are obtained with small sample size case groups relative to the large amount of data. However, in selecting the target, the small group which is going to be studied, it must be selected with very definite criteria and as few variables as possible.

In regards to the target peaks determined, these must be validated with a broader case group and bioinformatic tools must be used, effectively [22]. The plentiful candidate proteins available consist mostly of proteins which are the response of the acute phase, and cancer specific proteins have not yet gained clinical validity. The main advantages and disadvantages of each technique have been outlined in Table **1**.

### 2.1 Breast Cancer Clinical Applications

#### 2.1.1 Tissue Microarray Applications

The first protein array study in breast cancer was published by Kononen in 1998 [23]. This technique has been used to research the clinical significance of a small number of target proteins, frequently in large series of specimens. To date, (2009) there are 843 pubmed sourced publications on the use of tissue microarray applications for breast cancer diagnosis and follow up.

#### 2.1.2 Use for Diagnostic Purposes

##### 2.1.2.1 Molecular Level Definition Of Previously Known Diagnostic Classes

The genetic classification in breast cancer has been defined as sporadic (approximately 90% of cases) and hereditary (approximately 10% of cases) [25]. Afterwards, it has been differentiated according to their mutation conveyance in the BRCA1 and BRCA2 genes. This genotypic classification has been confirmed by Hedenfalk *et al*. [24] using a DNA microarray with a series of genes also containing the CCND1 gene. Palacios *et al.* [26] have confirmed this molecular classification with a study (CCND1, hormone receptors, p53, ERBB2, cell cycle regulators, apoptosis and basal cell indicator proteins) at the protein level covering 37 proteins. BRCA2 cancers have been found to be related to cycle regulators, D type cyclines (D1, D3), and CDK4. However, it has also been reported that in BRCA1 cancers, the ER/ERBB2 negativity, rapid proliferation, and basal phenotype is widespread [26, 27]. In the recognition of the molecules of lobular and ductal cancers, the use of the proteins EMP1, DVL1, DDR1 and PRKC1 is recommended together with E-cadherin [28].

Medullar breast carcinoma is a rare cancer but is known to have a poor prognosis. The persistence of the difficulties with morphological diagnosis has required the molecular definition. A definition using a series containing 18 proteins [29] has been confirmed by Bertucci *et al.* with their study using a DNA microarray [30]. At the protein level, an increase in p-cadherin, M1B1/Ki67, negative ERBB2, and positive p53 have been linked with medullar breast carcinoma.

In the studies which bring clarity to advances in breast oncogenesis, it has been observed that the role of the protein 14-3-3σ, described as a tumour suppressor in previous publications, is less than had been supposed [31], and the lymph node metastasis has been linked to ERBB2 status [32]. 

Another cancer, whose diagnosis involves difficulty, is the inflammatory breast cancer. Very few things are known concerning the molecular structure of this cancer which is rare but can frequently be fatal. In proteomic studies, with a protein signature defined by an increase in E-cadherin, ER(-), MIB1(+), MUC1(cytoplasmic staining) and ERBB2(+), 91% of the cancer can been defined [33-35].

##### 2.1.2.2 Discrimination of New Subgroups in Breast Cancer and Definition at the Molecular Level

In the general hierarchical classification of breast cancer there are many studies defining the molecular structures of the ER positive and ER negative groups. With the addition of proteins related to cell type and signal pathway to these defined structures, five molecular subtypes of ER positive tumours have been distinguished. The sub types defined at the proteomic level, luminal A, luminal B, basal, overexpressing ERBB2, and normal, have been confirmed by clinical data [36-39].

Together with the pathologic characteristics of breast cancer, in studies which are conducted on large sample series defining the molecular sub types, as many as 97 proteins have been determined, including ER, PR, ERBB2, p53, CK5/6, CK8/18, cyclin E, Ki67, BCL2, cyclin D1, and E-cadherin [40-42]. In the evaluations performed in conjunction with survival periods, 26 proteins were selected for follow up and the 5 year survival period was determined to be 80%. In addition to these proteins, with an investigation performed on four separate groups [metabolic (ER status), functional (proliferation, mitosis, differentiation)] protein patterns and survival expectations were classified as 3 different sub groups [A1, A2, B] [43]. These studies revealed more than ten protein markers that may distinguish disease subgroups clinically and biologically more reliably than the prognostic markers were being used [44, 45].

A defined molecular classification can be performed for luminal and ERBB2 positive tumours with proteomic studies, whether for diagnostic or therapeutic purposes. However, a valid diagnostic and therapeutic molecular definition for the basal group has not yet been obtained. In a small number of studies ER, ERBB2 negativity, CK5, CK5/6, EGFR positivity has been proposed as a diagnostic marker for a small sample group. This molecular pattern has been evaluated together with survival in a larger sample group [46-49].

##### 2.1.2.3 Proteomic Microarray in Breast Cancer Prognosis

The majority of the studies that are done in this field, have been realized and oriented towards scanning only one protein during the cancer follow up, in large sample groups. In studies performed with multiple protein patterns, AIB1, MUC1, MUC3, COX2, TERT, EpCAM/TACSTD1, EPIL, in the basal group crystalline α-B, cytokeratin 5 and 17, annexin A8, have been linked with poor clinical trend, whereas STAT5, BCL2, in the ER-luminal group GATA3, in the ERBB2 group GATA4, Ki67, ERBB2, have been linked with favourable clinical trend [50-62].

It was reported by Bertucci *et al.* [63] that a 21 protein pattern defined in a 5 year metastasis prediction of survival study concluded more favourably than the current standards, and in an independent study 9 of these proteins were approved [64, 65].

##### 2.1.2.4 Tissue Microarray Studies in the Prediction of Therapeutic Response

There are very few tissue microarray studies for therapeutic response in breast cancer. Simon *et al.* [66] proposed the use of KIT mutations for imatinib, Linke *et al.* [67] proposed the use of BCL2, ERBB2, MYC, TP53 proteins for tamoxifen and Rouzier *et al.* [68] proposed the use of TAU protein for paklitaksel.

Imatinib therapy’s target KIT expression was not associated with survival. Unlike fragile histidine triad, tyrosine-phosphorylated STAT5, or BCL2 proteins correlated with favorable clinical outcome. BCL2 was the only independent prognostic factor. BCL2 impact was then confirmed in an independent series of 1,961 tumors [64].

The favorable prognostic impact of GATA3 expression was correlated at the protein level [59].

Genes included in the basal cluster were also tested, and their negative prognostic role was confirmed in several hundreds of tumors; crystallin alpha B, cytokeratins 5, 17 and annexin A8. Using DNA microarrays identified a gene expression signature correlated with ERBB2 status [63].

The protein level the positive correlation of GATA4 and Ki67 with ERBB2. Rouzier *et al.* [68] confirmed on 122 independent tumors the correlation between low expression of the microtubule-associated protein TAU and sensitivity to neoadjuvant paclitaxel identified by gene profiling. 

ER status, the 21-protein pattern was the strongest independent predictor of clinical outcome. Supervised analysis was applied to nine proteins of 21 proteins pattern in stage I to III breast cancers treated with adjuvant tamoxifen [67].

### 2.2 Mass Spectroscopy Applications

In the years between January 1995 and November 2009 a total of 1191 articles were published concerning the application of mass spectrometry in breast cancer. Only 20 of these studies involved protein definition [69].

#### 2.2.1 Use of Mass Spectroscopy for Diagnostic Purposes

To establish and define protein patterns in the clinical meaning, elegant studies have been conducted, mostly with the MALDI/SELDI methods in numerous biological sources such as serum, plasma, tissue interstitial fluid, thin needle aspiration fluid, ductal lavage fluid, and saliva.

Despite being a less commonly chosen source in clinical studies due to the difficulty in tissue sampling, because of the diagnostic biomarkers peculiar to cancer, tumour tissue is a very good source. However, due to some reasons such as the difference in protein content validation of the compounds for breast cancer has not been obtained, yet. Umar *et al.* [70] have reported 9 tryptic peptides, which was determined as specific to breast cancer but was not defined structurally, as a diagnostic biomarker. Sanders *et al.* [71] defined S100-A6 protein, as a growth factor in breast tumour, for being an increasing marker, and the proteins S100-A8 and ubiquitin as a reducing marker. 

As is the case for all forms of cancer, serum and plasma are the preferred sources in biomarker studies that are performed for breast cancer because; they produce a rather active response to the physiological and pathological processes of the human body. This is a rich source of information on proteins. Together with the proteins secreted by the tumour, this also includes in the proteins of the normal tissue and plasma which is destroyed by proteases specific to the tumour, and those proteins causing a general response or a response local to the tumour. Despite the difficulty arising from this complex structure, methods ease in sampling and repetability makes it extremely suitable [72, 73].

In serum and plasma sourced studies performed using the MALDI-TOF-MS and SELDI-TOF-MS methods, mostly without having a structural definition, protein patterns have been proposed for purposes of diagnosis and classification in breast cancer. Becker *et al.* [74] in the definition of cancers involving BRCA-1 mutation, Laronga *et al.* [75] and Vlahou *et al.* [76] have proposed the use of protein patterns for diagnosis and classification. However, the structural definitions of proteins have not been made in these studies, and their validity has not yet been tested in an independent case group. In the case of seven protein patterns proposed in a diagnosis and classification which is carried out by Belluco *et al.* [77] although they have not been structurally defined, their validity has been tested in an independent case group and they have been presented as candidate proteins.

Three protein pattern biomarkers have been determined by Li *et al.* in breast cancer, one reducing (4.3 kDa), and two increasing (8.1 and 8.9 kDa) and in subsequent studies, their structural definitions have been determined respectively as ITIH4 (inter-alpha-tyripsin inhibitor heavy chain H4), C3a_desArgΔ8_ (C3a des-arginine-C terminal truncated peptide), and C3a_desArg_ (C3a des-arginine) and these have been repeated in independent case groups [78, 79].

However, in later publications related to these biomarkers; the increase in cancer cases of the 8.1kDa marker has not been found to be meaningful [80-83], and the 8.9kDa marker is reported to have decreased in metastatic recurrences [84]. As for the ITIH4 (4.3kDa) fragment, in studies performed by Song *et al.* [85], Villenueva *et al.* [86] and Fung *et al.* [87] it has been found to increase in cancer cases. Thus in the diagnosis of breast cancer the value of these 3 biomarkers proposed by Li *et al.* is still in dispute.

As ITIH4, the use of markers such as fibrinopeptide A, fibrinogen alpha, C3f, C4a, apolipoprotein A-IV, bradykinin, factor XIII, and transthyretin, reflecting the clotting status in the blood of cancer sufferers, are suggested for the diagnostic and classification purposes. The serum and plasma levels of these markers imply variations peculiar to the matrix [87, 88].

A very few of the studies made on ductal lavage fluid and needle aspiration fluid, traditionally used for cytological evaluations, have a normalisation based on protein content been performed. For this reason, they have revealed a broad distribution. Sauther *et al.* [89, 90] found 3 protein peaks, one of them being the haemoglobin beta chain isoform. In another study of very low sample number, an increase in neutrophile peptides has been demonstrated [91].

A matrix has been constructed for breast cancer biomarkers in saliva secretions. It was found that soluble c-erbB-2 and CA 15.3, and 5 proteins of high molecular weight (18, 113, 170, 228, and 287 km/z), as yet without a structural definition, increased in cancer cases [92].

##### 2.2.1.1 Use of Mass Spectroscopy in Follow up Of Breast Cancer

Proteomic studies in the follow up of breast cancer, together with use for diagnostic purposes, are still limited in number. A pattern defined as 40 protein signatures proposed by Goncalves *et al.* [93], predicted the clinical results of 83% of patients, correctly. This pattern, which includes haptoglobin alpha-1, complement C3a, transferin, and apoliprotein A-I and C-I, has not yet been confirmed by an independent group. 

In tissue lysates, an increase in ubiquitin and a decrease in ferritin light chain have been linked with good clinical progress [94], and have been confirmed in independent groups and cell series [95-97]. Studies performed on cerebral and spinal cord fluid are important from the aspect of metastases within the central nervous system. Central nervous system metastases have been defined with 77% accuracy using numerous peptides such as apoliprotein A-1 and E, haptoglobulin, transferin, transthyretin, prostaglandin D2 synthase, and haemopexin [98, 99].

The clinical progress of cancer is predicted according to TNM classification. In microarray based studies 5 different molecular type subgroups have been determined (luminal A and B, ERBB2 overexpressing, basal-like, and normal-like), and confirmed with tissue protein arrays [68,100-102].

Heat shock protein (HSP) 27 and annexin V increase in luminal A type cancers. It has been determined that S100-A9 increases in basal type cancers and it has been linked with poor clinical progress [95].

##### 2.2.1.2 Use of Mass Spectroscopy in Prediction of Therapeutic Response

The correct prediction of chemo-sensitivity in cancer therapy, whilst offering protection from toxic side effects, will cause a reduction in the use of ineffective medication and improved clinical results. For the determination of biomarkers which will provide prediction of therapeutic response and follow up of therapy, in the studies performed on drug sensitive and drug resistant (doxorubicin and paclitaxel) breast cancer cells with SELDI-TOF-MS, a large number of structurally undefined protein peaks have been proposed. Dowling *et al.* [103], proposed the use of transferrin fragments, linked with poor clinical progress, for the prediction of paclitaxel resistance. In treatments stimulating apoptosis, a decrease in ubiquitin and S100-A6 and abnormal expression in breast cancer tissue was found [94,104].

However, the results of these studies have not yet been transferred to clinical samples. In the limited number of in vivo studies performed in this field, it was determined that the kininogen and apolipoprotein A-II decreased in the shock table caused by docetaxel [105], and the structurally undefined SELDI peak, determined as 2790 m/z, increased markedly after paclitaxel infusion administered as an adjuvant [106].

However, it has not yet been possible to define a proteomic predictive marker to predict either therapeutic response or the responses to cytotoxic treatment, whether of micro-metastatic carcinomas or of the whole body.

## 3 CONCLUSION

The proteomic methods are being used in breast cancer researches and in recent years at an increasing rate. In the literature (pubmed) between the years 1996 and 2009, there are about 556 publications related to breast cancer and proteomics. In these studies examined by the code updating committee of ASCO (the American Society of Clinical Oncology) which regulates the use of tumour markers in breast cancer, many candidate proteins, found [107]. 

In serum, aspiration fluid (NAF), tumour tissue and intercellular fluid (TIF), were evaluated as proteomic indicators showing promise for clinical use. However, it was also proposed that these researches should be repeated with prospective studies to be performed with well-defined larger sampling groups, in different populations and with different analytical methods. In clinical trials, there are about 40 studies in progress relating to breast cancer and proteomics. At present, none of the proteomic profiling techniques has been validated sufficiently for use in patient care [107-109].

In the definition of cancer indicators, the proteomic technologies are producing very valuable data, differentiating and defining functional and regulatory pathways, determining the structure of disease causing molecules in tissue and biologic fluids, and manifest the disease stages or differences specific to the disease or to the individual.

This data will be reflected to the clinic in the form of the definition of effective markers providing early disease diagnosis, and the detection of response to treatment and of post treatment relapse.

In spite of all these encouraging developments, the usage of the proteomic markers for diagnostic purposes still requires a series of validation studies. As a consequence of all these studies, the use in conjunction with other diagnostic procedures (mammography, immunoassay, ultrasound, faecal occult blood test, etc.), early diagnosis, and also the follow up of responses to treatment may become important.

In conclusion, a proteomic marker valid for clinical use has not yet been defined in breast cancer [107]. However, the proteomic studies, which gained pace with the founding in 2001 of the international Human Proteome Organisation (HUPO), have shown promising results from the point of view of early breast cancer diagnosis, follow up and therapeutic predictive markers.

## Figures and Tables

**Table 1:** The Main Advantages and Disadvantages of Proteomic Techniques

Technique	Advantages	Disadvantages

Protein Microarray
	High throughput	Detects only known proteins
	Specific for protein interactions with many class of molecules	Antibody specificity
		Difficult to have protein in native conformation

2D-Page
	Hundreds to thousands of polypeptides can be analyzed in a single run	Manual work required
	The proteins can be separated in pure form	Problematic analysis of very low- or very high-Mr proteins
	Polypeptides can also be probed with antibodies and tested for post-translational modifications.	Salt ions could interfere with protein separation
	Used to study differential expression of proteins between cell types	Suppression of signal by high abundant proteins
	Semi-quantitative measurement of protein expression	Limited reproducibility
	MS identifies peptides originating mainly from a single protein; identification of the protein more direct	Smaller dynamic range than some other separation methods

Mass Spectroscopy	High sensitivity	MS identify peptides; final software analysis groups peptides belonging to a protein; identification of proteins less direct
	Automation is developed,	Manual work is required
	High throughput experiments	Low protein identification rate (<10%)
	Suitable for coupling with other methods	No match with experimentally measured Mr and pH

MALDI-TOF-MS	High sensitivity	Requires relatively pure samples
“Matrix-assisted laser desorption ionization”	Relatively quick analysis	More ambiguity in identifications due to lack of true sequence dependence of
		Exact, or high homology protein sequence must be present in database

SELDI-TOF-MS	High sensitivity	Requires validation for clinical diagnosis
“Surface-enhance laser desorption ionization”	High throughput	Relatively expensive
	Suitable for clinical samples	A selection process prior to data analysis of the spectra which has poor quality
	Automation is developed, a little manual work is required	
